# Editorial: Cardiovascular responses and diseases at high altitude

**DOI:** 10.3389/fcvm.2022.1024263

**Published:** 2022-09-14

**Authors:** Lan Huang

**Affiliations:** ^1^Institute of Cardiovascular Diseases of PLA, The Second Affiliated Hospital, Third Military Medical University (Army Medical University), Chongqing, China; ^2^Department of Cardiology, The Second Affiliated Hospital, Third Military Medical University (Army Medical University), Chongqing, China

**Keywords:** high altitude, cardiovascular, acute mountain sickness (AMS), heart rate variability, pulmonary artery pressure

The wonder and beauty of high altitude, such as the Himalayas, the Alps, and the Andes, etc., are attractive to a growing number of travelers, workers, and pilgrims. However, unacclimatized individuals may suffer from acute mountain sickness (AMS), high-altitude pulmonary edema (HAPE), or high-altitude cerebral edema (HACE). In order to facilitate the life-sustaining oxygen delivery to the main organs, such as heart, brain, kidney and so on, cardiovascular system plays a crucial role in adaptation to the bad environment of high altitude, including hypobaric hypoxia, low temperature, low humidity and high radiation. However, under certain conditions, the failure of cardiovascular adaptations to acute or chronic high altitude exposure is sometimes associated with several disadvantageous outcomes, including increase of proarrhythmic risk, systemic hypertension, high altitude pulmonary hypertension, right ventricular (RV) hypertrophy, RV failure and so on. Focusing on this topic, we would like to introduce the recent published articles in the section “*Cardiovascular responses and diseases at high altitude*” on Frontiers in Cardiovascular Medicine.

Growing evidences showed that the incidence of AMS was associated with cardiovascular responses under acute HA exposure. It had been reported that acute HA exposure might result in significant alterations in heart rate variability (HRV), which might be associated with the occurrence of AMS. However, Boos et al. reported that increasing ultra-short HRV was affected in a similar manner to gold-standard 300 s but could not predict AMS. Thus, the association between HRV and AMS may be controversial. Nevertheless, Chen R. et al.'s recent research showed that acute HA exposure resulted in blood pressure (BP) variations, Excessive BP load variations were associated with AMS. Therefore, at least part of the cardiovascular responses may be associated with the occurrence of AMS.

Acute or chronic HA exposure induced hypoxic pulmonary vasoconstriction and/or remodeling, which resulted in elevation of pulmonary artery pressure (PAP). The increase of RV afterload as well as hypobaric hypoxia may lead to myocardial injury and alterations in cardiac function. Bian et al.'s recent research indicated that the baseline concentrations of Ang II and NO at sea level could independently predict the elevated PAP after acute HA exposure, which may represent part of the pathophysiological mechanisms of HA-induced increase of PAP. Moreover, the effects of HA exposure on cardiac function were still unclear. Chen X. et al.'s research showed that RV systolic function was impaired in recent (<1 year) migrants to high altitude but improved during the long-term dwelling. LV remodeling persists in long-term migrants (>5 years) but without impairment of LV systolic or diastolic function (Chen X. et al.). These results described the characteristics for the effects of HA exposure on the heart. Additionally, A combination model was developed for the prediction of cardiorespiratory fitness impairment at high altitude, this model was consisted of post-submaximal exercise SpO_2_ at SL and the presence of EPAS1 rs13419896-A and EGLN1 rs508618-G variants (Yang et al.).

Although great advances have been achieved with regard to the influence of high altitude exposure on cardiovascular system and the underlying mechanisms, major issues upon this topic are still largely unresolved. Firstly, a large scale of multi-national high altitude study is needed to explore the presence, prevalence, risk factors, and clinical outcomes of the major cardiovascular disorders; secondly, a long-term monitoring is warranted to confirm whether the observed effects under acute high altitude exposure is transient or persistent; thirdly, more randomized clinical trials should be performed to test the efficiency and safety of the potential drugs against the major cardiovascular disorders at high altitude; lastly, guidelines or expert consensus with regard to high altitude related cardiovascular disorders are expected to standardize the diagnosis and management.

## Author contributions

LH drafts the manuscript.

## Conflict of interest

The author declares that the research was conducted in the absence of any commercial or financial relationships that could be construed as a potential conflict of interest.

## Publisher's note

All claims expressed in this article are solely those of the authors and do not necessarily represent those of their affiliated organizations, or those of the publisher, the editors and the reviewers. Any product that may be evaluated in this article, or claim that may be made by its manufacturer, is not guaranteed or endorsed by the publisher.

